# Neural Correlates of Theory of Mind Are Preserved in Young Women With Anorexia Nervosa

**DOI:** 10.3389/fpsyg.2020.568073

**Published:** 2020-09-10

**Authors:** Monica Leslie, Daniel Halls, Jenni Leppanen, Felicity Sedgewick, Katherine Smith, Hannah Hayward, Katie Lang, Leon Fonville, Mima Simic, William Mandy, Dasha Nicholls, Declan Murphy, Steven Williams, Kate Tchanturia

**Affiliations:** ^1^Department of Psychological Medicine, Institute of Psychiatry, Psychology & Neuroscience (IoPPN), King’s College London, London, United Kingdom; ^2^Department of Psychology, University of Chester, Chester, United Kingdom; ^3^School of Education, University of Bristol, Bristol, United Kingdom; ^4^Department of Forensic and Neurodevelopmental Sciences, Institute of Psychiatry, Psychology & Neuroscience (IoPPN), King’s College London, London, United Kingdom; ^5^Department of Psychology, Institute of Psychiatry, Psychology & Neuroscience (IoPPN), King’s College London, London, United Kingdom; ^6^Division of Brain Sciences, Imperial College London, London, United Kingdom; ^7^South London and Maudsley NHS Foundation Trust, London, United Kingdom; ^8^Research Department of Clinical, Health and Educational Psychology, University College London, London, United Kingdom; ^9^Division of Psychiatry, Imperial College London, London, United Kingdom; ^10^Department of Neuroimaging, Institute of Psychiatry, Psychology and Neuroscience (IoPPN), King’s College London, London, United Kingdom; ^11^Department of Psychology, Ilia State University, Tbilisi, Georgia

**Keywords:** anorexia nervosa, theory of mind, autism spectrum disorder, neuropsychology, functional magnetic resonance imaging

## Abstract

People with anorexia nervosa (AN) commonly exhibit social difficulties, which may be related to problems with understanding the perspectives of others, commonly known as Theory of Mind (ToM) processing. However, there is a dearth of literature investigating the neural basis of these differences in ToM and at what age they emerge. This study aimed to test for differences in the neural correlates of ToM processes in young women with AN, and young women weight-restored (WR) from AN, as compared to healthy control participants (HC). Based on previous findings in AN, we hypothesized that young women with current or prior AN, as compared to HCs, would exhibit a reduced neural response in the medial prefrontal cortex (mPFC), the inferior frontal gyrus, and the temporo-parietal junction (TPJ) whilst completing a ToM task. We recruited 73 young women with AN, 45 WR young women, and 70 young women without a history of AN to take part in the current study. Whilst undergoing a functional magnetic resonance imaging (fMRI) scan, participants completed the Frith-Happé task, which is a commonly used measure of ToM with demonstrated reliability and validity in adult populations. In this task, participants viewed the movements of triangles, which depicted either action movements, simple interactions, or complex social interactions. Viewing trials with more complex social interactions in the Frith-Happé task was associated with increased brain activation in regions including the right TPJ, the bilateral mPFC, the cerebellum, and the dorsolateral prefrontal cortex. There were no group differences in neural activation in response to the ToM contrast. Overall, these results suggest that the neural basis of spontaneous mentalizing is preserved in most young women with AN.

Anorexia nervosa (AN) is a severe eating disorder characterized by food restriction and compensatory behaviors leading to body weight which is excessively low for the individual’s height and development status (American Psychiatric Association, 2013). AN has a complex etiology, with a number of genetic and environmental risk factors contributing to onset of the disorder. Recent theoretical models have highlighted the importance of interpersonal difficulties in contributing to the onset and maintenance of AN (Schmidt and Treasure, 2006).

Theory of mind (ToM) has been defined as the ability to infer information about others’ emotions, intentions, knowledge, and beliefs from social interactions or given information (Frith and Frith, 2005). ToM abilities are therefore critical in most social situations to effectively understand and respond to the behaviors and intentions of others. Problems in ToM have been well-documented in autism and recent research has also found problems in ToM among people with AN, including difficulties with emotional and cognitive ToM (Bora and Köse, 2016; Leppanen et al., 2018; Kerr-Gaffney et al., 2019; Sedgewick et al., 2019). It is possible that ToM processes may hinder individuals’ response to talking therapies, such as by contributing to poor self-insight, and may impact affected individuals’ ability to access and utilize social support networks in the recovery process (Bora and Köse, 2016). It is, therefore, pertinent to better characterize the nature of ToM difficulties in AN and its underlying biological mechanisms in order to better understand the development of AN and possible social-cognitive targets for treatment intervention (Russell et al., 2009).

In the general population, ToM is associated with a complex network of brain regions. In particular, the temporo-parietal junction (TPJ) has been highlighted as a putative region that supports the formation of mental representations (Abu-Akel and Shamay-Tsoory, 2011; Döhnel et al., 2012). Following initial detection and representation of mental states, previous authors have hypothesized that the TPJ subsequently relays this information via the superior temporal sulcus (STS) to limbic and paralimbic regions for emotional processing (Abu-Akel and Shamay-Tsoory, 2011; Gao et al., 2019).

The bulk of neuroimaging research administering a ToM task during scanning has found different patterns of activation in autistic people^1^ compared to control participants. For example, research has found lower levels of activation in the right TPJ (Castelli et al., 2002; Kirkovski et al., 2016) and altered functional connectivity between anterior and posterior brain regions among autistic people during ToM tasks (Kana et al., 2009). However, more recent evidence has suggested that these differences may be specific to men, with autistic women exhibiting similar activation in the right TPJ and ventromedial prefrontal cortex (mPFC) during a mentalizing task compared to that of typically developing women (Kirkovski et al., 2016; Lai et al., 2019).

By contrast, recent evidence has highlighted differences in the brain networks recruited during ToM tasks in women with AN versus healthy controls (HCs), which may underpin functional differences in ToM abilities. McAdams and Krawczyk (2011), for example, found that, when compared to the HC participants, participants with a history of AN exhibited lower neural activation in brain regions forming part of the social cognition network, including the right inferior frontal gyrus, the bilateral TPJ, and the left fusiform gyrus during an implicit social attribution task. Schulte-Rüther et al. (2012) conducted a functional magnetic resonance imaging (fMRI) study using a similar ToM task in female adolescent inpatients with AN and HC participants. The authors found reduced neural activation in the middle and anterior temporal cortex and mPFC during the ToM task, as compared to the HC group. Furthermore, the level of hypoactivation in the mPFC was correlated with clinical outcome 1 year following discharge.

The current study aimed to expand on previous neuroimaging research into ToM in AN in a more highly powered study, thus enabling us to draw more confident conclusions about the degree of difference in the neural underpinning of ToM in young women with AN, and those weight-restored from AN, as compared to age-matched controls. We also sought to investigate the relationship between ToM-related neural activation and autistic features in young women with AN. Whilst undergoing an fMRI scan, participants completed the Frith-Happé task, which is a commonly used measure of ToM with demonstrated reliability and validity (Abell et al., 2000; White et al., 2011). Based on previous findings in AN, we hypothesized that the mPFC, the inferior frontal gyrus, and the TPJ would be associated with a reduced blood-oxygenated-level-dependent (BOLD) response in young women with, and weight-restored from, AN as compared to HCs, whilst completing the Frith-Happé task. We also hypothesized that greater levels of autistic characteristics in participants with AN would be associated with reduced ToM-related neural activation in the mPFC and related circuits extending to the TPJ.

## Materials and Methods

### Participants

A total of 188 young women between 16 and 25 years old participated in the current study. Seventy-three women met DSM-5 criteria for AN at the time of the study, 23 women were weight-restored from AN but exhibited continuing elevated levels of eating disorder symptoms, 22 women were in full recovery from AN, and 70 comparison women had no current or prior history of an eating disorder. Given the low sample sizes for the weight-recovered and fully recovered participant samples, these groups’ data were pooled into a single weight-recovered (WR) participant group for all analyses. The BMI range of HC participants was 18.29–33.39, the BMI range of participants with acute AN (AAN) was 12.65–18.50, and the BMI range of WR participants was 18.36–26.81. The average duration of illness for participants with current AN was 3.10 years (*SD* = 2.56 years) and the average duration of illness for WR participants was 4.53 years (*SD* = 2.78 years). Demographic statistics and clinical characteristics associated with each participant sample are presented in Table 1. Full inclusion and exclusion criteria for the study and details of the participants’ medication use are presented in the Supplementary Material.

**TABLE 1:** Descriptive demographic and clinical statistics.

	Healthy Control (*n* = 70)			Acute AN (*n* = 67)			Weight-restored AN (*n* = 49)			K–W Test Statistic	*P*
	Mean (SD)	Median (IQR)	Skew	Mean (SD)	Median (IQR)	Skew	Mean (SD)	Median (IQR)	Skew		
Age (Years)	19.64 (3.30)	18.54 (17.39–22.72)	0.52	18.70 (2.78)	18.40 (16.53–20.90)	0.44	19.72 (3.27)	18.94 (17.31–22.41)	0.23	3.85	0.278
BMI	22.82 (3.31)	22.26 (20.68–24.35)	1.03	16.61 (1.41)	16.82 (15.77–17.77)	−0.65	20.84 (2.26)	19.96 (19.17–21.87)	1.20	123.81	<0.001
IQ	109.05 (6.86)	110.35 (104.78–113.66)	−0.85	111.55 (7.79)	110.77 (106.22–117.17)	−0.06	111.80 (7.52)	111.18 (107.46–117.38)	−0.63	4.63	0.201
EDE-Q Global Score	0.60 (0.83)	0.30 (0.14–0.60)	2.82	3.33 (1.47)	3.62 (2.14–4.55)	−0.46	2.83 (1.67)	2.97 (1.13–4.30)	−0.25	81.41	<0.001
AQ10	2.31 (1.72)	2.00 (1.00–3.00)	0.95	3.98 (2.41)	4.00 (2.00–6.00)	0.36	3.54 (2.16)	3.00 (2.00–5.00)	0.75	20.81	<0.001

*AN = anorexia nervosa; AQ10 = Autism Quotient-10 item version; BMI = body mass index; EDE-Q = Eating Disorder Examination – Questionnaire version; K–W = Kruskal–Wallis; IQ = intelligence quotient; IQR = interquartile range.*

Participants with AAN were recruited from the South London and Maudsley National Health Service Foundation Trust. The HC and WR participant groups were recruited via social media, via the website for BEAT (the UK’s charity for eating disorders), and through advertisements in the local community. All participants provided written informed consent to take part in the study and, for participants under the age of 18, parental consent was also obtained. Ethical approval for the study was granted by the London – Surrey Borders Research Ethics Committee (REC Reference: 17/LO/0271). All study activities were in completed in accordance with the Declaration of Helsinki.

### Measures

The Eating Disorders Examination – Questionnaire version (Fairburn et al., 2009), the Hospital Anxiety and Depression Scale (Zigmond and Snaith, 1983), the National Adult Reading Test (Nelson, 1982), Autism Quotient-10 item version (Allison et al., 2012), and the Autism Diagnostic Observation Schedule (Lord et al., 2000) were administered to the participants. Details about these measures are presented in the Supplementary Material.

#### The Frith-Happé Animations

The Frith-Happé animations depict a series of cartoons in which a red triangle and a blue triangle can be seen to move around a central open box, often in a way that implies they are animate and interacting (Abell et al., 2000). The Frith-Happé animations fall into three categories: (1) Random movement, in which the two triangles appear to float across the screen, occasionally bumping into each other, but displaying no symbolic social interaction; (2) Goal-directed movement, in which the triangles move in the same direction, and may appear to chase each other, but do not exhibit mentalizing behavior; and (3) ToM interactions, in which the triangles appear to take the other shape’s thoughts and beliefs into account, such as by tricking or coaxing the other triangle to do something. The Frith-Happé animations are sensitive to difficulties with ToM even in autistic people with an IQ within the normal range, who pass standard first- and second-order false belief tasks (Abell et al., 2000). The Frith-Happé animations have a standardized coding system that produces an accuracy measure and a language measure for each of the three types of trials. Each trial’s accuracy is rated as 0 if the participant’s narrative contains a plainly wrong description and/or focuses on an unimportant aspect, 1 if the participant’s narrative contains a partial description of the sequence, but is imprecise or incomplete, and 2 if the participant’s narrative is a spot-on description of the story or the actions represented. Each trial’s language was coded as 0 if the participant describes a simple action with no interaction between the triangles, 1 if the participant describes interaction between the triangles without reference to mental states, and 2 if the participant uses mental state verbs to describe reciprocal interactions between the triangles.

### Procedure

Each participant attended two study sessions. During the first session, participants completed the self-report questionnaires and structured clinical interviews (e.g., the ADOS). Participants were screened for MRI safety prior to proceeding to the second session.

Upon presentation to the second study session, participants completed a narrative version of the Frith-Happé animations outside of the scanner. During each trial of the Frith-Happé animations, the participants were asked to describe what they thought the triangles were doing. Participants’ descriptions of each trial were audio-recorded and these behavioral data, collected outside of the scanner, were later analyzed. Participants subsequently underwent an fMRI scan in which they completed a battery of neuropsychological tests. The Frith-Happé task was repeated inside the scanner as before except that, rather than the participant describing what the triangles were doing, at the end of each trial participants were instead asked to use a button box to indicate whether the triangles had exhibited random, goal-directed, or ToM movements. The multiple-choice version of the Frith-Happé animations has previously been validated in adults within the context of fMRI scanning paradigms (White et al., 2011).

### fMRI Scan Acquisition

A total 307 volumes were acquired during the Frith-Happé task. Images within the fMRI scans were acquired with a slice thickness of 4 mm and a slice gap of 0.5 mm. A total of 28 slices were acquired in a top to bottom order. The field of view was 192 mm^2^ with a 64 × 64 matrix size. The resulting voxel size was therefore 3 mm × 3 mm × 4 mm. The scan was conducted with an echo time of 30 ms and a repetition time of 2,000 ms. The flip angle was set to 80 degrees. A 3D high- spatial-resolution, Magnetization-Prepared Rapid Acquisition (3D MPRAGE) T1-weighted scan was also acquired. Field of view was 270 mm^2^, TR/TE/TI = 7.312/3.016/400 ms. Two dummy scans were acquired at the start of the task and were subsequently discarded.

### Statistical Analysis

#### Behavioral Data Analysis

The audio recording of each trial in the Frith-Happé task was coded by one researcher, and then checked by a separate researcher. Initial inter-rater reliability was 92.23%. Discrepancies were subsequently reviewed by the lead author, such that instances of agreement with the second coder were confirmed and instances of disagreement were resolved by discussion. We planned to compare the accuracy and language scores of the three participants groups for random, goal-directed, and ToM trials on the Frith-Happé task using between-groups ANOVAs in line with the analyses previously conducted by Abell et al. (2000). However, as the residuals for the between-groups ANOVAs were not normally distributed, we instead conducted a Kruskal–Wallis test for each comparison and subsequently controlled for multiple corrections using an alpha rate of *p* < 0.05_FWE–corrected_.

#### MRI Data Pre-processing

We conducted pre-processing of the MRI data using fMRIprep 1.2.6-1 (Esteban et al., 2017, 2019), which is based on Nipype 1.1.7 (Gorgolewski et al., 2011; Gorgolewski, 2017). The full boilerplate associated with fMRIPrep, containing extensive details of pre-processing, is presented in the Supplementary Material.

#### MRI Data Analysis

We conducted both first- and second-level processing using FSL FEAT (FMRI Expert Analysis Tool) Version 6.00 (Smith et al., 2004; Jenkinson et al., 2012). At the single subject level, the data were modeled using the general linear model framework. We operationalized the “ToM” regressor as a linear contrast increasing in value from random trials, to goal-directed movements, to ToM trials and a separate contrast decreasing in social value from ToM trials, to goal-directed movements, to random trials. The BOLD signal was modeled by convolving our design matrix with a Double Gamma function. We included global signal, derivatives of motion parameters, squares of motion parameters, and a scrubbing variable excluding volumes with a framewise displacement >0.9 as confound variables at the single-subject level.

At the group level, we conducted region-of-interest (ROI) analyses using FSL featquery. We constructed the ROI masks based on peak coordinates from previous relevant studies. ROIs were 10 mm spheres based on coordinates identified by previous ToM research for the right inferior frontal gyrus [MNI coordinates [52 28 8] (McAdams and Krawczyk, 2011)] and the right TPJ [MNI coordinates [54, −52, 26] (Krall et al., 2015)]. As a 10 mm sphere localized in the mPFC crossed the brain boundary, we instead constructed a 9 mm spherical mask within the mPFC in order to avoid extracting null data from outside of the brain [MNI coordinates [4 60 20] (Schulte-Rüther et al., 2012)]. We subsequently conducted exploratory whole brain analyses using cluster level inference with a cluster threshold of *Z* > 3.1 and *p* < 0.05, corrected for multiple comparisons using Gaussian random field theory.

Four participants did not complete the ToM task and two participants had scans of unusable quality, resulting in a total of 182 participants’ data included in the final analysis. A power analysis conducted in G^∗^Power revealed that our between-groups analyses were powered to detect small to medium effect sizes (*f* = 0.23) (Erdfelder et al., 1996).

## Results

### Behavioral Data Analyses

Descriptive statistics associated with the accuracy and language scores of the Random, Goal-Directed, and ToM trials for each participant group and results of the Kruskal–Wallis tests comparing the participant groups are presented in Table 2. There were no significant between-group differences in accuracy for any of the three trial types. The analysis initially identified differences in the level of social language used for the random and ToM trials, such that HC participants tended to use greater levels of social language to describe random trials than participants with AAN and WR participants tended to use greater levels of social language to describe ToM trials than participants with AAN. However, these differences did not survive correction for multiple comparisons.

**TABLE 2:** Descriptive statistics associated with the accuracy and language scores of the Random, Goal-Directed, and Theory of Mind trials for each participant group.

	Healthy Control M (SD)	Acute AN M (SD)	Weight-Restored AN M (SD)	Kruskal test statistic	*p*-value	FWE-corrected *p*-value
Random Accuracy	1.62 (0.612)	1.73 (0.477)	1.70 (0.434)	1.05	0.591	0.591
Random Language	0.53 (0.610)	0.23 (0.460)	0.42 (0.679)	9.47	0.009	0.052
Goal-Directed Accuracy	1.52 (0.311)	1.45 (0.320)	1.57 (0.308)	4.23	0.121	0.288
Goal-Directed Language	1.02 (0.169)	0.96 (0.119)	1.04 (0.193)	5.91	0.052	0.192
Theory of Mind Accuracy	1.25 (0.434)	1.12 (0.325)	1.24 (0.366)	4.47	0.107	0.288
Theory of Mind Language	1.32 (0.351)	1.21 (0.277)	1.37 (0.338)	7.06	0.029	0.138

*AN = anorexia nervosa; FWE = familywise error.*

### ROI Analyses

We conducted a between-groups ANOVA comparing mean BOLD activation within the mPFC, the TPJ and the inferior frontal gyrus. There were no significant differences between the three participant groups for any of the ROIs. We subsequently added psychiatric medication use as a covariate in a between-groups ANCOVA. This ANCOVA also did not reveal significant differences between the three participant groups for any of the ROIs.

### Exploratory Whole-Brain Analyses

An initial one-sample *t*-test revealed 19 significant clusters associated with increasing complexity of the ToM contrast and a separate one-sample *t*-test revealed 20 significant activation clusters associated with decreasing complexity of the ToM contrast. These task-activated regions conform with previous norms reported within the ToM literature, including activation within the TPJ, mPFC, and inferior frontal gyrus. The full results of these one-sample *t*-tests are presented in Supplementary Tables 1, 2.

A between-groups ANOVA comparing the ToM contrast between the three participant groups did not reveal any significant clusters associated with increasing or decreasing social complexity of the ToM contrast. We next conducted a sensitivity analysis excluding participants taking psychoactive medication to account for any suppression of between-group differences driven by psychotropic medication. This between-groups ANOVA also failed to detect any significant between-groups differences associated with increasing or decreasing complexity of the ToM contrast.

Finally, we conducted exploratory whole brain analyses within the AAN participant group including the AQ10, ADOS Communication and Social subscale, ADOS interaction subscale, ADOS imagination and creativity subscale, the ToM accuracy and language scores, BMI, global EDE score, and illness duration as covariates in nine separate one-sample *t*-tests. The ADOS communication and social subscale and the ADOS interaction subscale were both correlated with BOLD response to decreasing complexity of the ToM contrast within the right extrastriate cortex (i.e., higher ADOS scores were associated with lower BOLD response to ToM trials). Cluster peaks for the ADOS interaction subscale were located at MNI coordinates [23.5, −78.5, −10.5] and [15.5, −82.5, −16.5]. The cluster peak for the ADOS Communication and Social subscale was located at MNI coordinate [23.5, −78.5, −12.5]. The ToM imagination and creativity subscale was associated with decreasing complexity of the ToM contrast within the left dorsal posterior cingulate cortex. The cluster peak was located atMNI coordinate [−6.5, −42.5, 41.5]. Illness duration was correlated with the BOLD response to increasing complexity of the ToM contrast in the left parahippocampal gyrus, MNI coordinate [−22.5, −22.5, −14.5] and to decreasing complexity of the ToM contrast in the left premotor cortex, MNI coordinates [−22.5, −4.5, 55.5] and [−26.5, −0.5, 63.5]. There were no significant associations between any of the other covariates and BOLD response to the ToM contrast amongst participants with current AN.

## Discussion

The current study aimed to test for differences in the brain correlates of ToM processing in young women with AN, young women weight-restored from AN, and healthy comparison participants. We hypothesized that participants with, or weight-restored from, AN would exhibit reduced activation in the mPFC, the TPJ, and the inferior frontal gyrus in response to a ToM task, when compared to those without history of an eating disorder. Our manipulation check revealed that task-activated regions conformed with previous norms reported within the ToM literature, including activation within the TPJ, mPFC, and inferior frontal gyrus. However, the data did not support any of these hypotheses, as there were no significant between-group differences in BOLD response to a spontaneous mentalizing task. We also hypothesized that neural activation within the mPFC, the TPJ, and the inferior frontal gyrus would be negatively correlated with autistic traits amongst participants with AN. The latter hypothesis was not supported by the results, as autistic traits were not associated with task-related activation in these three hypothesized regions. However, the ADOS communication and interaction scales were associated with task-related neural response in early visual processing regions. Furthermore, illness duration was found to be associated with task-related neural response in the left parahippocampal gyrus and left premotor cortex.

Our behavioral findings corresponded with previous studies which also found no evidence of differences in accuracy between women with a history of AN and HC participants on spontaneous mentalizing tasks (McAdams and Krawczyk, 2011; Schulte-Rüther et al., 2012). However, the lack of group differences in brain response to the ToM task was an unexpected result, which contrasts with previous studies finding altered patterns of BOLD responses to a very similar task among adult women in recovery from AN (McAdams and Krawczyk, 2011) and in a previous study conducted in adolescents with AN (Schulte-Rüther et al., 2012). There are several potential explanations for this difference in findings. First, it may be the case that differences in the neural underpinning of ToM develop progressively throughout the course of the illness and remain for some time after recovery, which might explain why a different pattern of neural response to a similar ToM task has previously been observed amongst older adult women in weight recovery from AN, as compared to age-matched control participants, but not in our sample of young adults with AN (McAdams and Krawczyk, 2011). However, this explanation does not account for the failure to replicate previously documented differences in BOLD responding to a ToM task amongst adolescents with AN (Schulte-Rüther et al., 2012).

It is possible that the present results may reflect no true differences in the neural underpinnings of ToM across the entire population of young adults with AN. Indeed, our relatively large sample size of 188 young adults, including 73 young adults with current AN, 45 young adults in weight recovery from AN, and 70 HC participants, is likely to be associated with more stable effect sizes and reduced confidence intervals than the previous study conducted in young people with AN, which recruited only 19 participants with current AN. This is consistent with the notion that ToM impairments are present in a subgroup of those with AN, but do not feature on average across cases with adolescent onset (Stewart et al., 2017).

The current results suggest that differences are specifically observed in individuals with AN who are high in autistic characteristics. That is, higher levels of communication and interaction difficulties were associated with increased neural response in the extrastriate cortex to decreasing complexity of the ToM contrast. This finding may be explained by previous research demonstrating that, in contrast to neurotypical participants, autistic participants demonstrate a lack of attentional modulation when viewing social stimuli, which is associated with differences in the activation of early visual regions, including the primary visual cortex and extrastriate cortex (Bird et al., 2006). Previous evidence suggests that this between-groups effect is particularly pronounced for subtle, versus overt, social cues (Zürcher et al., 2013), which are exemplified by the representational social cues depicted by triangles in the Frith-Happé task.

The association between duration of AN and task-related activation in the left parahippocampal gyrus and left premotor cortex is, however, more difficult to explain on the basis of previous literature in populations with AN. In the general population, parahippocampal gyrus activation has been observed in response to completing empathy and face recognition tasks (Völlm et al., 2006; van Veluw and Chance, 2014). It may be that the effects of more prolonged malnourishment disrupt circuits related to social memory and the perception of social stimuli mediated by the parahippocampal gyri. However, further evidence is needed to more clearly establish the functional significance of this finding.

The current findings add to our understanding of the complex pattern of differences exhibited by people with AN across different domains of ToM. A recent meta-analysis of ToM abilities in people with AN found that, while affected individuals exhibit statistically significant differences in the domains of emotional ToM, understanding simple social interactions, and understanding complex social interactions, there was no significant difference in the domain of implicit social attribution, measured in the current study (Leppanen et al., 2018).

Indeed, the extent of blanket differences in spontaneous mentalizing abilities and gross differences in associated neural activation has more recently been questioned, even in autistic populations. For example, a recent large study recruiting more than 300 autistic participants found no differences in performance or neural activation on the Frith-Happé task, when compared to HC participants (Moessnang et al., 2020). Furthermore, previous evidence, which did find differences in the neural correlates of ToM in autistic men, did not find similar differences in autistic women (Kirkovski et al., 2016; Lai et al., 2019). These previous findings are difficult to reconcile with our current observation that some components of autistic traits are, indeed associated with neural response to a ToM task. Further research in large samples of autistic women will help to clarify whether such differences may be specific to those with the greatest levels of communication and social interaction difficulties, as suggested by our current findings in young women with AN.

Strengths of the current study include the large sample size of women completing both behavioral and fMRI tasks, allowing greater confidence in the effect sizes found within the current set of analyses. However, this study is not without limitations, including the specific component of ToM measured within the Frith-Happé task. Thus, while the current study provided no evidence for differences in the brain underpinnings of spontaneous mentalizing in young women with a history of AN versus HCs, problems in this population have previously been observed for other components of ToM (Leppanen et al., 2018), and may be associated with a different pattern of neural activation on other ToM tasks. Additionally, it is possible that presenting a descriptive version of the task prior to the neuroimaging scan resulted in a “training” effect, perhaps resulting in the recruitment of a greater degree of memory processes and lesser degree of ToM processes than would have been observed had participants viewed the task for the first time during the fMRI scan. Further research will therefore be required to corroborate these results and examine potential differences in the neural underpinnings of emotional ToM and complex social interactions. Finally, as this study was conducted exclusively in young women, the current findings should not be generalized to men or older adults with AN.

While the current study has replicated consistent findings in brain regions that underpin ToM processing, including within the rTPJ and mPFC, we did not find evidence for between-group differences in the neural underpinnings of spontaneous mentalizing in young women with a history of AN versus HC participants. It should be noted that this null finding may be due, in part, to the specific ROI masks analyzed in the current study. We based our ROIs on previous studies conducted in AN to maximize applicability to the population recruited in the current study. However, these previous activation peaks were observed in relatively small samples, and a different pattern of results may have been observed had we based our ROIs on regions that are generally activated during the Frith-Happé task in HC participants. Future research will help to clarify whether different patterns of neural activation underpin behavioral performance in other domains of ToM and more clearly establish the functional significance of the association between illness duration and task-related neural response. Overall, the current set of findings suggests that the neural processing of spontaneous mentalizing remains more intact in young women with AN than previously thought.

## Data Availability Statement

The raw data supporting the conclusions of this article will be made available by the authors, without undue reservation.

## Ethics Statement

The studies involving human participants were reviewed and approved by The London – Surrey Borders Research Ethics Committee (REC Reference: 17/LO/0271). Written consent to participate was provided by participants. Written consent was also provided by the participant’s legal guardian where participants were under the age of 18.

## Author Contributions

KT, KL, JL, LF, MS, WM, DN, and SW contributed to the conceptualization and design of the study. JL and FS conducted the study. ML and DH conducted the analyses reported in the current study and wrote the current manuscript. JL, FS, and KS also contributed to data analysis. JL, FS, KS, HH, KL, LF, MS, WM, DN, DM, SW, and KT edited the current manuscript. All authors contributed to the interpretation of the results.

## Conflict of Interest

The authors declare that the research was conducted in the absence of any commercial or financial relationships that could be construed as a potential conflict of interest.

## Footnotes

1 Identity-first language (i.e., autistic person), opposed to person-first language (i.e., person with autism), is preferred by many autistic people and their allies. Therefore, in this article, the authors use predominantly identity-first language to describe this population (Kenny et al., 2016).
