# Corrigendum: Neural Correlates of Theory of Mind Are Preserved in Young Women With Anorexia Nervosa

**DOI:** 10.3389/fpsyg.2020.617275

**Published:** 2020-11-30

**Authors:** Monica Leslie, Daniel Halls, Jenni Leppanen, Felicity Sedgewick, Katherine Smith, Hannah Hayward, Katie Lang, Leon Fonville, Mima Simic, William Mandy, Dasha Nicholls, Declan Murphy, Steven Williams, Kate Tchanturia

**Affiliations:** ^1^Department of Psychological Medicine, Institute of Psychiatry, Psychology & Neuroscience (IoPPN), King's College London, London, United Kingdom; ^2^Department of Psychology, University of Chester, Chester, United Kingdom; ^3^School of Education, University of Bristol, Bristol, United Kingdom; ^4^Department of Forensic and Neurodevelopmental Sciences, Institute of Psychiatry, Psychology & Neuroscience (IoPPN), King's College London, London, United Kingdom; ^5^Department of Psychology, Institute of Psychiatry, Psychology & Neuroscience (IoPPN), King's College London, London, United Kingdom; ^6^Division of Brain Sciences, Imperial College London, London, United Kingdom; ^7^South London and Maudsley NHS Foundation Trust, London, United Kingdom; ^8^Research Department of Clinical, Health and Educational Psychology, University College London, London, United Kingdom; ^9^Division of Psychiatry, Imperial College London, London, United Kingdom; ^10^Department of Neuroimaging, Institute of Psychiatry, Psychology and Neuroscience (IoPPN), King's College London, London, United Kingdom; ^11^Department of Psychology, Ilia State University, Tbilisi, Georgia

**Keywords:** anorexia nervosa, theory of mind, autism spectrum disorder, neuropsychology, functional magnetic resonance imaging

In the original article, there was an error. The ADOS subscales included as covariates in exploratory fMRI analyses were mislabeled.

A correction has been made to *Results, Exploratory Whole-Brain Analyses, Paragraph 3*.

The corrected paragraph is presented below:

Finally, we conducted exploratory whole brain analyses within the AAN participant group including the AQ10, ADOS Communication and Social subscale, ADOS interaction subscale, ADOS imagination and creativity subscale, the ToM accuracy and language scores, BMI, global EDE score, and illness duration as covariates in nine separate one-sample *t*-tests. The ADOS communication and social subscale and the ADOS interaction subscale were both correlated with BOLD response to decreasing complexity of the ToM contrast within the right extrastriate cortex (i.e., higher ADOS scores were associated with lower BOLD response to ToM trials). Cluster peaks for the ADOS interaction subscale were located at MNI coordinates [23.5, −78.5, −10.5] and [15.5, −82.5, −16.5]. The cluster peak for the ADOS Communication and Social subscale was located at MNI coordinate [23.5, −78.5, −12.5]. The ToM imagination and creativity subscale was associated with decreasing complexity of the ToM contrast within the left dorsal posterior cingulate cortex. The cluster peak was located at MNI coordinate [−6.5, −42.5, 41.5]. Illness duration was correlated with the BOLD response to increasing complexity of the ToM contrast in the left parahippocampal gyrus, MNI coordinate [−22.5, −22.5, −14.5] and to decreasing complexity of the ToM contrast in the left premotor cortex, MNI coordinates [−22.5, −4.5, 55.5] and [−26.5, −0.5, 63.5]. There were no significant associations between any of the other covariates and BOLD response to the ToM contrast amongst participants with current AN.

The authors apologize for this error and state that this does not change the scientific conclusions of the article in any way. The original article has been updated.

