# Supplementary material for: Neural Correlates of Theory of Mind Are Preserved in Young Women With Anorexia Nervosa
Source: Front Psychol. 2020 Sep 10;11:568073. doi: 10.3389/fpsyg.2020.568073 (PMC7511528; doi:10.3389/fpsyg.2020.568073)
Supplement: Supplementary file 1 [file Data_Sheet_1.docx]

**Supplementary Material**

**Supplementary Table 1**

*Results of the One-sample t-test Exploratory Whole Brain Analysis for Neural Activation Associated with Increasing Complexity for the Theory of Mind Contrast*

A *Z* > 3.1 cluster-forming threshold was used. We report significant clusters at the

*p* < .05 threshold.

| Cluster Number | Hemisphere | k | p_FWE_ | Peak Coordinates | | | Description |
| --- | --- | --- | --- | --- | --- | --- | --- |
|  |  |  |  | **x** | **y** | **z** |  |
| Cluster 1 | Right | 10,911 | < .001 | 43.5 | -46.5 | -12.5 | Fusiform gyrus, posterior superior temporal gyrus, and the middle temporal area |
|  |  |  |  | 47.5 | -40.5 | 11.5 |  |
|  |  |  |  | 49.5 | -24.5 | -4.5 |  |
|  |  |  |  | 47.5 | -20.5 | -10.5 |  |
|  |  |  |  | 47.5 | -38.5 | 3.5 |  |
|  |  |  |  | 41.5 | -58.5 | -10.5 |  |
| Cluster 2 | Left | 8,531 | < .001 | -42.5 | -52.5 | -14.5 | Fusiform gyrus, visual association area, and the angular area |
|  |  |  |  | -28.5 | -98.5 | -8.5 |  |
|  |  |  |  | -24.5 | -100 | -2.5 |  |
|  |  |  |  | -22.5 | -102 | -6.5 |  |
|  |  |  |  | -58.5 | -52.5 | 13.5 |  |
|  |  |  |  | -46.5 | -64.5 | -14.5 |  |
| Cluster 3 | Right | 2,771 | < .001 | 49.5 | 23.5 | 23.5 | Dorsolateral prefrontal cortex and the inferior frontal gyrus |
|  |  |  |  | 39.5 | 7.5 | 27.5 |  |
|  |  |  |  | 51.5 | 19.5 | 29.5 |  |
|  |  |  |  | 35.5 | 9.5 | 29.5 |  |
|  |  |  |  | 47.5 | 11.5 | 29.5 |  |
|  |  |  |  | 57.5 | 25.5 | 27.5 |  |
| Cluster 4 | Right | 1,097 | < .001 | 21.5 | -60.5 | 25.5 | Dorsal posterior cingulate cortex, ventral posterior cingulate cortex, and the precuneus |
|  |  |  |  | 17.5 | -54.5 | 19.5 |  |
|  |  |  |  | 15.5 | -56.5 | 23.5 |  |
|  |  |  |  | 11.5 | -52.5 | 41.5 |  |
|  |  |  |  | 1.5 | -62.5 | 37.5 |  |
|  |  |  |  | 3.5 | -54.5 | 33.5 |  |
| Cluster 5 | Right | 223 | < .001 | 17.5 | -82.5 | -30.5 | The medial cerebellum |
|  |  |  |  | 23.5 | -82.5 | -32.5 |  |
|  |  |  |  | 13.5 | -74.5 | -28.5 |  |
|  |  |  |  | 25.5 | -74.5 | -34.5 |  |
| Cluster 6 | Left | 216 | < .001 | -20.5 | -60.5 | 23.5 | Visual association area and the precuneus |
|  |  |  |  | -16.5 | -66.5 | 35.5 |  |
| Cluster 7 | Bilateral | 149 | < .001 | -0.5 | 57.5 | 35.5 | The medial prefrontal cortex |
|  |  |  |  | -8.5 | 61.5 | 35.5 |  |
|  |  |  |  | -0.5 | 51.5 | 45.5 |  |
|  |  |  |  | 3.5 | 49.5 | 39.5 |  |
|  |  |  |  | -0.5 | 45.5 | 43.5 |  |
|  |  |  |  | 3.5 | 61.5 | 25.5 |  |
| Cluster 8 | Right | 120 | < .001 | 33.5 | -24.5 | 15.5 | Primary auditory cortex and the temporo-parietal junction |
|  |  |  |  | 37.5 | -26.5 | 21.5 |  |
| Cluster 9 | Left | 112 | < .001 | -34.5 | -28.5 | 17.5 | The temporo-parietal junction |
| Cluster 10 | Right | 108 | < .001 | 3.5 | 17.5 | 67.5 | The supplementary motor area |
|  |  |  |  | 7.5 | 9.5 | 73.5 |  |
|  |  |  |  | 1.5 | 11.5 | 63.5 |  |
|  |  |  |  | 9.5 | 23.5 | 65.5 |  |
|  |  |  |  | 11.5 | 5.5 | 73.5 |  |
| Cluster 11 | Right | 54 | < .001 | 39.5 | -78.5 | 39.5 | The angular area and the extrastriate cortex |
|  |  |  |  | 39.5 | -80.5 | 35.5 |  |
|  |  |  |  | 45.5 | -76.5 | 29.5 |  |
| Cluster 12 | Right | 52 | < .001 | 3.5 | -26.5 | 65.5 | The primary motor cortex |
| Cluster 13 | Right | 33 | .003 | 17.5 | -28.5 | 27.5 | White matter |
| Cluster 14 | Left | 32 | .004 | -22.5 | -24.5 | 5.5 | White matter |
|  |  |  |  | -20.5 | -24.5 | -0.5 |  |
| Cluster 15 | Right | 26 | .014 | 39.5 | -4.5 | 13.5 | Primary motor cortex |
| Cluster 16 | Right | 24 | .022 | 11.5 | -26.5 | -34.5 | The brainstem |
|  |  |  |  | 11.5 | -24.5 | -40.5 |  |
| Cluster 17 | Right | 24 | .022 | 41.5 | 11.5 | -20.5 | The temporopolar area |

**Supplementary Table 2**

*Results of the One-sample t-test Exploratory Whole Brain Analysis for Neural Activation Associated with Decreasing Complexity of the Theory of Mind Contrast*

A *Z* > 3.1 cluster-forming threshold was used. We report significant clusters at the

*p* < .05 threshold.

| Cluster Number | Hemisphere | k | p_FWE_ | Peak Coordinates | | | Description |
| --- | --- | --- | --- | --- | --- | --- | --- |
|  |  |  |  | **x** | **y** | **z** |  |
| Cluster 1 | Bilateral | 26,613 | < .001 | 15.5 | -78.5 | 9.5 | Right visual association area, right primary visual cortex, left visual association area, and the left primary visual cortex |
|  |  |  |  | 11.5 | -92.5 | 17.5 |  |
|  |  |  |  | -4.5 | -100 | 17.5 |  |
|  |  |  |  | -6.5 | -94.5 | 13.5 |  |
|  |  |  |  | -12.5 | -86.5 | 7.5 |  |
|  |  |  |  | -8.5 | -98.5 | 11.5 |  |
| Cluster 2 | Left | 869 | < .001 | -32.5 | 17.5 | 11.5 | The inferior frontal gyrus and the premotor cortex |
|  |  |  |  | -40.5 | 11.5 | 7.5 |  |
|  |  |  |  | -44.5 | 11.5 | 3.5 |  |
|  |  |  |  | -48.5 | -0.5 | 7.5 |  |
|  |  |  |  | -44.5 | 15.5 | -2.5 |  |
|  |  |  |  | -58.5 | 5.5 | 37.5 |  |
| Cluster 3 | Left | 856 | < .001 | -28.5 | 33.5 | 27.5 | Dorsolateral prefrontal cortex and the frontal eye fields |
|  |  |  |  | -26.5 | 39.5 | 43.5 |  |
|  |  |  |  | -30.5 | 37.5 | 35.5 |  |
|  |  |  |  | -32.5 | 43.5 | 39.5 |  |
|  |  |  |  | -30.5 | 33.5 | 47.5 |  |
|  |  |  |  | -22.5 | 31.5 | 11.5 |  |
| Cluster 4 | Right | 711 | < .001 | 31.5 | 17.5 | 11.5 | Inferior frontal gyrus, premotor cortex, and posterior superior temporal gyrus |
|  |  |  |  | 39.5 | 15.5 | 3.5 |  |
|  |  |  |  | 45.5 | 3.5 | 7.5 |  |
|  |  |  |  | 43.5 | 15.5 | -0.5 |  |
|  |  |  |  | 53.5 | -2.5 | -0.5 |  |
|  |  |  |  | 51.5 | 3.5 | -0.5 |  |
| Cluster 5 | Left | 393 | < .001 | -14.5 | -22.5 | 43.5 | Supplementary motor area and dorsal posterior cingulate cortex |
|  |  |  |  | -10.5 | -20.5 | 47.5 |  |
|  |  |  |  | -6.5 | -22.5 | 49.5 |  |
|  |  |  |  | -2.5 | -32.5 | 37.5 |  |
|  |  |  |  | -18.5 | -34.5 | 41.5 |  |
|  |  |  |  | -12.5 | -16.5 | 43.5 |  |
| Cluster 6 | Right | 229 | < .001 | 19.5 | 5.5 | 27.5 | White matter |
|  |  |  |  | 19.5 | -4.5 | 29.5 |  |
|  |  |  |  | 19.5 | -8.5 | 29.5 |  |
|  |  |  |  | 19.5 | 15.5 | 23.5 |  |
|  |  |  |  | 23.5 | -12.5 | 35.5 |  |
|  |  |  |  | 23.5 | 5.5 | 41.5 |  |
| Cluster 7 | Left | 177 | < .001 | -44.5 | -66.5 | -38.5 | Lateral cerebellum |
|  |  |  |  | -44.5 | -50.5 | -34.5 |  |
|  |  |  |  | -38.5 | -44.5 | -32.5 |  |
|  |  |  |  | -50.5 | -60.5 | -38.5 |  |
|  |  |  |  | -44.5 | -46.5 | -38.5 |  |
|  |  |  |  | -44.5 | -64.5 | -44.5 |  |
| Cluster 8 | Left | 80 | < .001 | -48.5 | -58.5 | 41.5 | The angular area |
|  |  |  |  | -42.5 | -52.5 | 35.5 |  |
|  |  |  |  | -44.5 | -52.5 | 39.5 |  |
|  |  |  |  | -50.5 | -54.5 | 49.5 |  |
| Cluster 9 | Right | 51 | < .001 | 35.5 | -44.5 | -30.5 | Lateral cerebellum |
|  |  |  |  | 37.5 | -54.5 | -30.5 |  |
|  |  |  |  | 33.5 | -52.5 | -28.5 |  |
| Cluster 10 | Left | 51 | < .001 | -12.5 | 39.5 | 23.5 | Medial prefrontal cortex and the dorsal anterior cingulate cortex |
|  |  |  |  | -8.5 | 37.5 | 13.5 |  |
|  |  |  |  | -10.5 | 41.5 | 17.5 |  |
|  |  |  |  | -12.5 | 41.5 | 7.5 |  |
|  |  |  |  | -14.5 | 37.5 | 13.5 |  |
| Cluster 11 | Right | 50 | < .001 | 5.5 | 53.5 | -0.5 | Medial prefrontal cortex |
|  |  |  |  | 13.5 | 59.5 | 1.5 |  |
|  |  |  |  | 11.5 | 55.5 | 1.5 |  |
| Cluster 12 | Left | 49 | < .001 | -36.5 | 49.5 | -10.5 | Frontopolar cortex |
|  |  |  |  | -28.5 | 45.5 | -10.5 |  |
| Cluster 13 | Left | 49 | < .001 | -30.5 | -48.5 | -48.5 | Inferior cerebellum |
|  |  |  |  | -26.5 | -40.5 | -46.5 |  |
| Cluster 14 | Left | 29 | .007 | -18.5 | -52.5 | -46.5 | Inferior cerebellum |
| Cluster 15 | Right | 28 | .009 | 33.5 | -34.5 | 11.5 | White matter |
|  |  |  |  | 29.5 | -26.5 | 9.5 |  |
| Cluster 16 | Right | 23 | .029 | 13.5 | -36.5 | 15.5 | White matter |
|  |  |  |  | 7.5 | -34.5 | 11.5 |  |
|  |  |  |  | 3.5 | -30.5 | 15.5 |  |
| Cluster 17 | Left | 22 | .037 | -40.5 | -48.5 | 1.5 | White matter |
|  |  |  |  | -42.5 | -40.5 | -6.5 |  |
| Cluster 18 | Right | 22 | .037 | 23.5 | -54.5 | 35.5 | White matter |
|  |  |  |  | 19.5 | -52.5 | 41.5 |  |
| Cluster 19 | Left | 21 | .048 | 43.5 | -36.5 | -8.5 | White matter |
|  |  |  |  | 43.5 | -42.5 | -4.5 |  |
| Cluster 20 | Left | 21 | .048 | -8.5 | -68.5 | -34.5 | Medial cerebellum |
|  |  |  |  | -6.5 | -72.5 | -38.5 |  |

*Inclusion and Exclusion Criteria for the Study*

Participants in the current AN participant group were required to meet DSM-5 criteria for AN at the point of recruitment and have a BMI less than 90% of the median BMI for age and gender or a body mass index (BMI) less than 18.5. Participants in the weight-recovered AN group must have previously been diagnosed with AN, but have had a BMI within the healthy weight range (18.5-25) during the 12-month period prior to study participation. Participants in the healthy control group were required to have no current eating disorder or history of an eating disorder. Participants in the healthy control group were also required to have a BMI within the healthy weight range (18.5-25). Exclusion criteria for the study included any neurological impairment (e.g., epilepsy), serious brain injury or learning difficulties, and MRI incompatibility (e.g., pregnancy, claustrophobia, inability to lie down flat, and any metal in or on the body which could not be removed).

*Participant Medication Use*

Thirty-eight women with current AN, 14 weight-restored women, 13 women in recovery from AN, and 13 healthy control women were taking medication at the time of the study. This amounted to 23% of the total sample taking some form of medication at the time of the study, less than half of whom were taking psychiatric medication (9.6% of the total sample).

With regards to psychiatric medication, 17 women with current AN were taking an antidepressant, 3 were taking an antipsychotic, 5 were taking both an antidepressant and an antipsychotic, and 1 was taking an antidepressant and benzodiazepine. Of the women recovered from AN, 5 were taking an antidepressant at the time of the study. Of the women who were weight-recovered from AN, 8 were taking an antidepressant, 1 was taking an antipsychotic, and 3 were taking an antidepressant and an antipsychotic. Two of the healthy control women were taking antidepressants and one healthy control women was taking a stimulant for attention deficit hyperactivity disorder (ADHD) at the time of the study.

*Measures*

*Eating Disorders Examination – Questionnaire version.* The Eating Disorder Examination – Questionnaire version (EDE-Q) is a self-report measure of eating disorder psychopathology. The EDE-Q assesses the raw frequency of common eating disorder behaviours and also contains four eating disorder psychopathology subscales measuring Restraint, Eating Concern, Weight Concern, and Shape Concern. Each subscale is presented in the form of a 7-point Likert scale. For each item, participants are asked to indicate over what range of days they exhibited each component of eating disorder psychopathology, where responses are anchored from 0 (“No days”) to 6 (“Every day”). Higher scores on the EDE-Q therefore indicate greater levels of eating disorder psychopathology. The EDE-Q is associated with acceptable criterion validity, with significantly different mean scores for each subscale among individuals with, versus without, a current eating disorder (18).

*Hospital Anxiety and Depression Scale*. The Hospital Anxiety and Depression Scale (HADS) is a 14-item self-report questionnaire assessing levels of depression and anxiety. Each item is presented on a 4-point Likert scale anchored from 0-3. The HADS yields separate anxiety and depression subscales, where higher scores on each subscale indicate greater levels of anxiety and depression, respectively. The HADS is associated with good concurrent validity, with strong positive correlations to other measures of anxiety and depression (20).

*The National Adult Reading Test.* The National Adult Reading Test (NART) is a measure of premorbid intellectual function in English-speaking adults. The test consists of a list of 50 written words with irregular spellings, which the participant is prompted to read aloud. The participants’ ability to pronounce each word correctly tests the participants’ vocabulary, which is used as a proxy measure for intelligence. Scores on the NART are converted to an estimated intelligence quotient (IQ) score. The NART exhibits good concurrent validity, with a strong positive correlation to scores on the Wechsler Adult Intelligence Scale (22). The primary advantage of administering the NART, as opposed to the WAIS, is that it takes a fraction of the time to complete, thus reducing participant burden.

*The Autism Quotient-10 item version.* The Autism Quotient-10 item version (AQ-10) is a 10-item questionnaire assessing autistic symptomatology. Items are presented in the form of a 4-point Likert scale, anchored from “strongly disagree” to “strongly agree”. Items are scored as either 0 or 1 depending on the direction of endorsement. Each item score is subsequently summed, such that higher scores on the AQ-10 indicate greater levels of autistic symptomatology. The AQ-10 has good sensitivity (88%) and specificity (91%) in the prediction of autism spectrum disorders (ASD) with a cut-off point of 6.0.

*The Autism Diagnostic Observation Schedule.* The Autism Diagnostic Observation Schedule (ADOS) is a semi-structured interview measuring autistic traits in the domains of social interaction, communication, play, and imaginative use of materials. Higher ratings within each module indicate greater levels of autistic traits. Each module has good test-retest reliability and excellent inter-rater reliability. The ADOS is associated with excellent sensitivity (82-95%) and specificity (80-100%) to detect ASD (20-22).

*fMRIprep Boilerplate*

Results included in this manuscript come from preprocessing performed using fMRIPrep 1.5.1rc1 (Esteban, Markiewicz, et al. (2018); Esteban, Blair, et al. (2018); RRID:SCR_016216), which is based on Nipype 1.3.0-rc1 (Gorgolewski et al. (2011); Gorgolewski et al. (2018); RRID:SCR_002502).

*Anatomical data preprocessing.* The T1-weighted (T1w) image was corrected for intensity non-uniformity (INU) with N4BiasFieldCorrection (Tustison et al. 2010), distributed with ANTs 2.2.0 (Avants et al. 2008, RRID:SCR_004757), and used as T1w-reference throughout the workflow. The T1w-reference was then skull-stripped with a Nipype implementation of the antsBrainExtraction.sh workflow (from ANTs), using OASIS30ANTs as target template. Brain tissue segmentation of cerebrospinal fluid (CSF), white-matter (WM) and gray-matter (GM) was performed on the brain-extracted T1w using fast (FSL 5.0.9, RRID:SCR_002823, Zhang, Brady, and Smith 2001). Brain surfaces were reconstructed using recon-all (FreeSurfer 6.0.1, RRID:SCR_001847, Dale, Fischl, and Sereno 1999), and the brain mask estimated previously was refined with a custom variation of the method to reconcile ANTs-derived and FreeSurfer-derived segmentations of the cortical gray-matter of Mindboggle (RRID:SCR_002438, Klein et al. 2017). Volume-based spatial normalization to one standard space (MNI152NLin2009cAsym) was performed through nonlinear registration with antsRegistration (ANTs 2.2.0), using brain-extracted versions of both T1w reference and the T1w template. The following template was selected for spatial normalization: ICBM 152 Nonlinear Asymmetrical template version 2009c [Fonov et al. (2009), RRID:SCR_008796; TemplateFlow ID: MNI152NLin2009cAsym].

Functional data preprocessing

*Functional data preprocessing.* For each of the 1 BOLD runs found per subject (across all tasks and sessions), the following preprocessing was performed. First, a reference volume and its skull-stripped version were generated using a custom methodology of fMRIPrep. The BOLD reference was then co-registered to the T1w reference using bbregister (FreeSurfer) which implements boundary-based registration (Greve and Fischl 2009). Co-registration was configured with six degrees of freedom. Head-motion parameters with respect to the BOLD reference (transformation matrices, and six corresponding rotation and translation parameters) are estimated before any spatiotemporal filtering using mcflirt (FSL 5.0.9, Jenkinson et al. 2002). BOLD runs were slice-time corrected using 3dTshift from AFNI 20160207 (Cox and Hyde 1997, RRID:SCR_005927). The BOLD time-series, were resampled to surfaces on the following spaces: fsaverage5. The BOLD time-series (including slice-timing correction when applied) were resampled onto their original, native space by applying a single, composite transform to correct for head-motion and susceptibility distortions. These resampled BOLD time-series will be referred to as preprocessed BOLD in original space, or just preprocessed BOLD. The BOLD time-series were resampled into standard space, generating a preprocessed BOLD run in MNI152NLin2009cAsym space. First, a reference volume and its skull-stripped version were generated using a custom methodology of fMRIPrep. Several confounding time-series were calculated based on the preprocessed BOLD: framewise displacement (FD), DVARS and three region-wise global signals. FD and DVARS are calculated for each functional run, both using their implementations in Nipype (following the definitions by Power et al. 2014). The three global signals are extracted within the CSF, the WM, and the whole-brain masks. Additionally, a set of physiological regressors were extracted to allow for component-based noise correction (CompCor, Behzadi et al. 2007). Principal components are estimated after high-pass filtering the preprocessed BOLD time-series (using a discrete cosine filter with 128s cut-off) for the two CompCor variants: temporal (tCompCor) and anatomical (aCompCor). tCompCor components are then calculated from the top 5% variable voxels within a mask covering the subcortical regions. This subcortical mask is obtained by heavily eroding the brain mask, which ensures it does not include cortical GM regions. For aCompCor, components are calculated within the intersection of the aforementioned mask and the union of CSF and WM masks calculated in T1w space, after their projection to the native space of each functional run (using the inverse BOLD-to-T1w transformation). Components are also calculated separately within the WM and CSF masks. For each CompCor decomposition, the k components with the largest singular values are retained, such that the retained componentsâ time series are sufficient to explain 50 percent of variance across the nuisance mask (CSF, WM, combined, or temporal). The remaining components are dropped from consideration. The head-motion estimates calculated in the correction step were also placed within the corresponding confounds file. The confound time series derived from head motion estimates and global signals were expanded with the inclusion of temporal derivatives and quadratic terms for each (Satterthwaite et al. 2013). Frames that exceeded a threshold of 0.5 mm FD or 1.5 standardised DVARS were annotated as motion outliers. All resamplings can be performed with a single interpolation step by composing all the pertinent transformations (i.e. head-motion transform matrices, susceptibility distortion correction when available, and co-registrations to anatomical and output spaces). Gridded (volumetric) resamplings were performed using antsApplyTransforms (ANTs), configured with Lanczos interpolation to minimize the smoothing effects of other kernels (Lanczos 1964). Non-gridded (surface) resamplings were performed using mri_vol2surf (FreeSurfer).

Many internal operations of fMRIPrep use Nilearn 0.5.2 (Abraham et al. 2014, RRID:SCR_001362), mostly within the functional processing workflow. For more details of the pipeline, see the section corresponding to workflows in fMRIPrep documentation.

*Copyright Waiver*

The above boilerplate text was automatically generated by fMRIPrep with the express intention that users should copy and paste this text into their manuscripts unchanged. It is released under the CC0 license.

**References**

Abraham, Alexandre, Fabian Pedregosa, Michael Eickenberg, Philippe Gervais, Andreas Mueller, Jean Kossaifi, Alexandre Gramfort, Bertrand Thirion, and Gael Varoquaux. 2014. “Machine Learning for Neuroimaging with Scikit-Learn.” Frontiers in Neuroinformatics 8. https://doi.org/10.3389/fninf.2014.00014.

Avants, B.B., C.L. Epstein, M. Grossman, and J.C. Gee. 2008. “Symmetric Diffeomorphic Image Registration with Cross-Correlation: Evaluating Automated Labeling of Elderly and Neurodegenerative Brain”. Medical Image Analysis 12 (1): 26-41. https://doi.org/10.1016/j.media.2007.06.004.

Behzadi, Yashar, Khaled Restom, Joy Liau, and Thomas T. Liu. 2007. “A Component Based Noise Correction Method (CompCor) for BOLD and Perfusion Based fMRI”. NeuroImage 37 (1): 90-101. https://doi.org/10.1016/j.neuroimage.2007.04.042.

Cox, Robert W., and James S. Hyde. 1997. “Software Tools for Analysis and Visualization of fMRI Data”. NMR in Biomedicine 10 (4-5): 171-78. https://doi.org/10.1002/(SICI)1099-1492(199706/08)10:4/5<171::AID-NBM453>3.0.CO;2-L.

Dale, Anders M., Bruce Fischl, and Martin I. Sereno. 1999. “Cortical Surface-Based Analysis: I. Segmentation and Surface Reconstruction”. NeuroImage 9 (2): 179-94. https://doi.org/10.1006/nimg.1998.0395.

Esteban, Oscar, Ross Blair, Christopher J. Markiewicz, Shoshana L. Berleant, Craig Moodie, Feilong Ma, Ayse Ilkay Isik, et al. 2018. “FMRIPrep”. Software. Zenodo. https://doi.org/10.5281/zenodo.852659.

Esteban, Oscar, Christopher Markiewicz, Ross W Blair, Craig Moodie, Ayse Ilkay Isik, Asier Erramuzpe Aliaga, James Kent, et al. 2018. “fMRIPrep: A Robust Preprocessing Pipeline for Functional MRI”. Nature Methods. https://doi.org/10.1038/s41592-018-0235-4.

Fonov, VS, AC Evans, RC McKinstry, CR Almli, and DL Collins. 2009. “Unbiased Nonlinear Average Age-Appropriate Brain Templates from Birth to Adulthood”. NeuroImage 47, Supplement 1: S102. https://doi.org/10.1016/S1053-8119(09)70884-5.

Gorgolewski, K., C. D. Burns, C. Madison, D. Clark, Y. O. Halchenko, M. L. Waskom, and S. Ghosh. 2011. “Nipype: A Flexible, Lightweight and Extensible Neuroimaging Data Processing Framework in Python”. Frontiers in Neuroinformatics 5: 13. https://doi.org/10.3389/fninf.2011.00013.

Gorgolewski, Krzysztof J., Oscar Esteban, Christopher J. Markiewicz, Erik Ziegler, David Gage Ellis, Michael Philipp Notter, Dorota Jarecka, et al. 2018. “Nipype”. Software. Zenodo. https://doi.org/10.5281/zenodo.596855.

Greve, Douglas N, and Bruce Fischl. 2009. “Accurate and Robust Brain Image Alignment Using Boundary-Based Registration”. NeuroImage 48 (1): 63-72. https://doi.org/10.1016/j.neuroimage.2009.06.060.

Jenkinson, Mark, Peter Bannister, Michael Brady, and Stephen Smith. 2002. “Improved Optimization for the Robust and Accurate Linear Registration and Motion Correction of Brain Images”. NeuroImage 17 (2): 825-41. https://doi.org/10.1006/nimg.2002.1132.

Klein, Arno, Satrajit S. Ghosh, Forrest S. Bao, Joachim Giard, Yrjö Häme, Eliezer Stavsky, Noah Lee, et al. 2017. “Mindboggling Morphometry of Human Brains”. PLOS Computational Biology 13 (2): e1005350. https://doi.org/10.1371/journal.pcbi.1005350.

Lanczos, C. 1964. “Evaluation of Noisy Data”. Journal of the Society for Industrial and Applied Mathematics Series B Numerical Analysis 1 (1): 76-85. https://doi.org/10.1137/0701007.

Power, Jonathan D., Anish Mitra, Timothy O. Laumann, Abraham Z. Snyder, Bradley L. Schlaggar, and Steven E. Petersen. 2014. “Methods to Detect, Characterize, and Remove Motion Artifact in Resting State fMRI”. NeuroImage 84 (Supplement C): 320-41. https://doi.org/10.1016/j.neuroimage.2013.08.048.

Satterthwaite, Theodore D., Mark A. Elliott, Raphael T. Gerraty, Kosha Ruparel, James Loughead, Monica E. Calkins, Simon B. Eickhoff, et al. 2013. “An improved framework for confound regression and filtering for control of motion artifact in the preprocessing of resting-state functional connectivity data”. NeuroImage 64 (1): 240-56. https://doi.org/10.1016/j.neuroimage.2012.08.052.

Tustison, N. J., B. B. Avants, P. A. Cook, Y. Zheng, A. Egan, P. A. Yushkevich, and J. C. Gee. 2010. “N4ITK: Improved N3 Bias Correction.” IEEE Transactions on Medical Imaging 29 (6): 1310-20. https://doi.org/10.1109/TMI.2010.2046908.

Zhang, Y., M. Brady, and S. Smith. 2001. “Segmentation of Brain MR Images Through a Hidden Markov Random Field Model and the Expectation-Maximization Algorithm”. IEEE Transactions on Medical Imaging 20 (1): 45-57. https://doi.org/10.1109/42.906424.
